# Pyknon-Containing Transcripts Are Downregulated in Colorectal Cancer Tumors, and Loss of *PYK44* Is Associated With Worse Patient Outcome

**DOI:** 10.3389/fgene.2020.581454

**Published:** 2020-11-12

**Authors:** Adriane Feijó Evangelista, Weder Pereira de Menezes, Gustavo Noriz Berardinelli, Wellington Dos Santos, Cristovam Scapulatempo-Neto, Denise Peixoto Guimarães, George A. Calin, Rui Manuel Reis

**Affiliations:** ^1^Molecular Oncology Research Center, Barretos Cancer Hospital, Barretos, Brazil; ^2^Department of Pathology, Barretos Cancer Hospital, Barretos, Brazil; ^3^Department of Endoscopy, Barretos Cancer Hospital, Barretos, Brazil; ^4^Translational Molecular Pathology Department, University of Texas MD Anderson Cancer Center, Houston, TX, United States; ^5^Life and Health Sciences Research Institute (ICVS), School of Medicine, University of Minho, Braga, Portugal; ^6^ICVS/3B's-PT Government Associate Laboratory, Guimarães, Portugal

**Keywords:** pyknons, non-coding RNAs, gene expression regulation, TP53 mutations, colorectal cancer, Brazilian population

## Abstract

Pyknons are specific human/primate-specific DNA motifs at least 16 nucleotides long that are repeated in blocks in intergenic and intronic regions of the genome and can be located in a new class of non-coding RNAs of variable length. Recent studies reported that pyknon deregulation could be involved in the carcinogenesis process, including colorectal cancer. We evaluated the expression profile of a set of 12 pyknons in a set of molecularly characterized colorectal cancer (CRC) patients. The pyknons (*PYK10*, *PYK14*, *PYK17*, *PYK26*, *PYK27*, *PYK40*, *PYK41*, *PYK42*, *PYK43*, *PYK44*, *PYK83*, and *PYK90*) expression was determined by qRT-PCR. A pilot analysis of 20 cases was performed, and consistent results were obtained for *PYK10*, *PYK17*, *PYK42*, *PYK44*, and *PYK83*. Further, the expression of the selected pyknons was evaluated in 73 CRC cases. Moreover, in 52 patients, we compared the expression profile in both tumor and normal tissues. All five pyknons analyzed showed significantly lower expression levels in the tumor compared to normal tissue. It was observed an association between expression of *PYK10* with *TP53* mutations (*p* = 0.029), *PYK17* to histologic grade (*p* = 0.035), and *PYK44* to clinical staging (*p* = 0.016). Moreover, levels of *PYK44* were significantly associated with the patient's poor overall survival (*p* = 0.04). We reported the significant downregulation of pyknons motifs in tumor tissue compared with the normal counterpart, and the association of lower *PYK44* expression with worse patient outcome. Further studies are needed to extend and validate these findings and determine the clinical-pathological impact.

## 1. Introduction

For a long time, the central dogma of molecular biology proposed RNA molecules to primarily be informational “messenger” between DNA and proteins (Sana et al., 2012; Cobb, 2015). However, only 2% of the total human genome sequence encodes genes (Mercer et al., 2009; Sana et al., 2012). Currently, it is known that the human transcriptome is more complicated than protein-coding gene collections and the intergenic regions (Pertea, 2012). Novel molecules have been discovered in the last years, and the new class of non-coding RNAs (ncRNAs) are divided into two main groups according to their nucleotide length, as small or long ncRNAs. These ncRNAs include microRNAs (miRNAs), Piwi-interacting RNAs (piRNAs), long intergenic non-coding RNAs (lincRNAs), transcription initiation RNAs (tiRNAs), sno-derived RNAs (sdRNAs), transfer RNA (t-RNA) fragments, long enhancer ncRNAs (eRNAs), and pyknon-containing transcripts among others (Pertea, 2012; Rigoutsos et al., 2017). These molecules are significant players of cellular transcription mechanisms (Cech and Steitz, 2014; Ling et al., 2015; Rigoutsos et al., 2017). The best-studied group among ncRNAs are microRNAs (miRNAs). These molecules are short RNAs of approximately 19–23 nucleotides (nt) in length that bind their target mRNAs in a sequence-dependent manner, regulating the expression of the corresponding protein-coding gene. Over the past decades, miRNAs have been found to act as mediators in molecular interactions and associated with countless diseases, including cancer (Calin et al., 2002; Bartel, 2009; Rigoutsos, 2009; Almeida et al., 2012; Tay et al., 2014; Amit et al., 2020).

Pyknons, derived from the Greek word meaning “dense, frequent, serried” was initially described by computational approaches, which aimed to investigate a high number of short sequence motifs across the human genome (Rigoutsos and Floratos, 1998; Rigoutsos et al., 2006). These motifs were posteriorly described to exhibit functional conservation in the absence of sequence conservation or synteny and at least one exonic instance in addition to the numerous identical instances in intergenic and intronic regions (Rigoutsos et al., 2006, 2017; Sana et al., 2012). Furthermore, pyknons show variable lengths and have been found in several long non-coding transcripts, suggesting that these motifs could tie together long non-coding transcripts and messenger RNAs on a broader network (Rigoutsos et al., 2017). Moreover, approximately 40% of known miRNAs show sequence similarity to pyknons, with similar transcript abundance in the cells, suggesting a putative link in such regulation process (Rigoutsos et al., 2006, 2017; Tsirigos and Rigoutsos, 2008). The previous study of pyknon sequences showed that these motifs are not syntenic, their sequences are organism-specific and are not conserved across genomes, and their intronic copies are over-represented in the same groups' protein-coding genes in primates and can be tissue-specific (Rigoutsos et al., 2006; Tsirigos and Rigoutsos, 2008; Robine et al., 2009). Despite a limited number of experiments, a possible clinical application of pyknons has been proposed as putative biomarkers due to their expression changes between health and disease, as candidates for therapeutic exploitation and also to act as a tool to discover new non-coding RNAs, that could be used to target specific transcripts in the tumor cell to increase the efficiency of immunotherapy (Rigoutsos et al., 2006; Dragomir et al., 2019). The most recent compendium of the available pyknons is organized in the pyknon database (http://cm.jefferson.edu/pyknons.html), which accounts for 209,432 distinct human pyknons at the moment and could be used to characterize these molecules and also the lncRNA interactions with other non-coding RNAs (Danis and Széll, 2018).

Colorectal cancers (CRC) constitute the third most diagnosed cancer worldwide and the second in mortality rates in 2018 (Bray et al., 2018). The last decades have witnessed an increase in CRC incidence, which can be justified by population aging, lifestyle factors like smoking, insufficient levels of physical activity, and poor dietary habits (Kuipers et al., 2015; Arnold et al., 2017). In Brazil, CRC is the second most common cancer type for both women and men in the Southeast region (INCA, 2020). The new Brazilian mortalities rate of CRC showed an increase for both men and women when comparing data between 1996 and 2012 (Oliveira et al., 2018). A recent study by our group characterized the CRC in the Brazilian population, showing high prevalence in the *APC* (71.4%), *TP53* (56.0%), *KRAS* (52.7%), *PIK3CA* (15.4%), and *FBXW7* (10.9%) gene drivers, together with alterations in the MAPK/ERK, PIK3/AKT, NOTCH, and receptor tyrosine kinase signaling pathways (Dos Santos et al., 2019). A better understanding of its biology and regulatory mechanisms are needed, including the understanding of the role of new transcripts.

Recently, Rigoutsos et al. (2017) performed a screening of pyknons that could be associated with colorectal cancer. They explored 11 pyknons located in regions associated with LOH and fragile-sites and identified a differential expression profile between normal and tumor tissue, reporting an association of pyknons expression with MSI status (*PYK14*, *PYK17*, *PYK40*, *PYK41*, and *PYK42*) and overall patient survival (*PYK90*) (Rigoutsos et al., 2017). Moreover, the authors showed that *PYK90* is a functional pyknon within a novel lncRNA (*N-BLR*) that regulates epithelial-to-mesenchymal transition (EMT) and promotes migration and invasion in colorectal cancer (Rigoutsos et al., 2017).

In the present study, we aimed to assess the expression levels of five selected pyknons from those mentioned above in a series of 73 molecularly characterized Brazilian colorectal cancer patients compared to the tumor with adjacent normal tissue in a subset of 52 cases. Besides, we associated the pyknons expression with patients' clinical, molecular, and genetic ancestry features.

## 2. Materials and Methods

### 2.1. Study Population and Tissue Sample Collection

This study analyzed 73 patients with colorectal cancer surgically treated between 2009 and 2013 at the Department of Colorectal Surgery of Barretos Cancer Hospital, Barretos, Brazil. Clinical and pathological features of patients, such as age, gender, primary tumor location, clinical staging, and histological grade, are summarized in Table 1. Overall, the age ranged from 29 to 89 years old (median 59 ± 14.25) with similar gender distribution. The stratification by age showed 37 (50.7%) patients in the adult group (20–59 years) and 36 (49.3%) in the group above 59 years. The sigmoid colon and rectum were the most prevalent primary sites, with 34.2% and 43.8%, respectively, and the majority were in clinical stage II (52.1%). Most of the patients were not submitted to radiotherapy (89.0%) or adjuvant chemotherapy (60.3%). The patients were followed for 80 months, and the median global survival for this cohort at 24 months was 85.6% after diagnosis. This series was previously reported regarding the molecular portrait of 150 cancer-related genes, MSI and genetic ancestry as European (median of 83.1%), followed by Native American (4.1%), Asian (3.4%), and African (3.2%) (Dos Santos et al., 2019). The samples comprised 73 fresh-frozen tumors and a subset of 52 paired normal adjacent tissue. Tissues were immediately snap-frozen following the specimen's excision at the surgery and stored at −80°C at the Barretos Cancer Hospital Biobank until processing. Slides from all tissue specimens were carefully macrodissected and subjected to histological examination to confirm the diagnostic. Only tumor samples with the presence of at least 60% of tumor cells were included. The Barretos Cancer Hospital Institutional Review Board approved this study (project nr # 684/2013).

**Table 1:** Clinicopathological and molecular features of Brazilian colorectal cancer patients.

**Characteristics**		****Frequency (*****n*****)****	****(%)****
**Median age (*****n*** **= 73)**	59 (±14.25)	–	–
**Age group**			
	<68 years	44	60.3
	≥ 68 years	29	39.7
**Gender**			
	Female	32	43.8
	Male	41	56.2
**Primary disease site**			
	Cecum	1	1.4
	Descendent colon	1	1.4
	Sigmoid colon	25	34.2
	Rectosigmoid junction	14	19.2
	Rectum	32	43.8
**Clinical staging (at diagnosis)**			
	0/I	16	21.9
	II (a-c)	38	52.1
	III (b-c)	15	20.5
	IV (b)	3	4.1
	NA	1	1.4
**Primary tumor (T)**			
	Tis/T1	12	16.4
	T2	14	19.2
	T3	39	53.4
	T4 (a,b)	7	9.6
	NA	1	1.4
**Regional lymph nodes (N)**			
	N0	51	69.9
	N1 (a,b,c)	14	19.1
	N2 (a,b)	5	6.8
	Nx	1	1.4
	NA	2	2.8
**Distant metastasis (M)**			
	M0	69	94.5
	M1a	3	4.1
	NA	1	1.4
**Histologic grade**			
	I	2	2.7
	II	67	91.8
	III	4	5.5
**Radiotherapy**			
	Yes	7	9.6
	No	65	89.0
	NA	1	1.4
**Adjuvant chemotherapy**			
	Yes	28	38.3
	No	44	60.3
	NA	1	1.4
MSI^*^			
	MSS + MSI-L	67	91.8
	MSI-H	3	4.1
	NA	3	4.1
APC^*^ mutation			
	Yes	42	57.5
	No	10	13.7
	NA	21	28.8
TP53^*^ mutation			
	Yes	37	50.7
	No	15	20.5
	NA	21	28.8
KRAS^*^ mutation			
	Yes	37	50.7
	No	33	45.2
	NA	3	4.1
PIK3CA^*^ mutation			
	Yes	7	9.6
	No	45	61.6
	NA	21	28.8
FBXW7^*^ mutation			
	Yes	5	6.8
	No	47	64.4
	NA	21	28.8
BRAF^*^ mutation			
	Yes	0	0.0
	No	69	94.5
	NA	4	5.5

* *Previous reported (Dos Santos et al., 2019); N/A = Not available*.

### 2.2. Nucleic Acid Isolation

Tumor nucleic acids were isolated from 25 mg of fresh-frozen tissue using Precellys ceramic beads in a lysis buffer for maceration, followed by QIAsymphony DNA Mini Kit following the Tissue 200 protocol (RNA extraction) for automated isolation in the QIAsymphony, according to the manufacturer's protocol (QIAGEN, Hilden, Germany). DNA and RNA concentration and quality were assessed by Nanodrop 2000 and Qubit (Thermo Scientific, Wilmington, DE, USA). The RNA integrity was assessed by the RNA Integrity Number (RIN) using Agilent 2100 Bioanalyzer (Agilent Technologies, Santa Clara, CA, USA), and only samples with RIN values ≥ 6.0 were considered.

### 2.3. Microsatellite Instability (MSI) Analyses

The MSI status was evaluated using a multiplex PCR comprising six quasi-monomorphic mononucleotide repeat markers (BAT-25, BAT-26, NR-21, NR-24, NR-27, and HSP110) and described previously (Dos Santos et al., 2019). Briefly, the analyses of MSI was performed using the GeneMapper v4.1 software (Applied Biosystems), and the status was classified as stable (MSS), when none of the markers were unstable, MSI-Low (MSI-L) when one of the markers were unstable or MSI-positive when two or more of the markers were unstable. MSS and MSI-L were considered MSI-negative (Berardinelli et al., 2018).

### 2.4. Mutation Status of *APC, TP53, KRAS, BRAF, PIK3CA*, and *FBXW7*

Our group recently reported the somatic mutation profile of 150 cancer-related genes in this cohort of colorectal cancer patients (Dos Santos et al., 2019). The mutation status was assessed using a Nextera Rapid Capture Custom Enrichment Kit, followed by Illumina HiSeq. Four thousand next-generation sequencing and the top mutated genes were selected to be associated with pyknons expression (Dos Santos et al., 2019). The most relevant hotspots regions of the genes *KRAS* (codons 12, 13, and 61) and *BRAF* (exons 11 and 15) were also validated by PCR followed by direct sequencing, as previously described by our group (Martinho et al., 2009; Yamane et al., 2014). The Sanger sequencing was performed in the 3500xL Genetic Analyzer (Applied Biosystems). Both sequences (forward and reverse) on electropherograms were analyzed visually, and all lesions with mutations were confirmed twice.

### 2.5. Quantitative Real Time-PCR (RT-qPCR)

The reverse transcription (RT) was performed using the Superscript II reverse transcriptase (Life Technologies). Then, the synthesized cDNA was amplified by quantitative real time-PCR (RT-qPCR), using KAPA SYBR FAST qPCR Kit Master Mix (2X) Universal (KAPA Biosynthesis). Target primers' sequences specific to each pyknon were the same described by Rigoutsos et al. (2017) and are shown in Supplementary Table 1. Every single reaction included 10.4 μL KAPA SYBR FAST qPCR Kit Master Mix (2X) Universal with ROX, 0.8 μL of the PCR primer set for an individual pyknon and 7.8 μL RNase free water. In these series, it was performed two technical replicates for all the samples. Real-time PCR was carried out in StepOne Real-time PCR System (Applied Biosystems) using the following cycling conditions: 95°C for 3 min, followed by 40 cycles of 95°C for 30 s, and 60°C for 20 s, followed by a hold at 4°C. The 2-ΔCt method was used to calculate the relative amount of each pyknon compared with the expression of the endogenous controls (average of *GAPDH*, *U6*, and *ACTB*). Similarly to Rigoutsos et al. (2017), if the expression values for the transcript of interest were not obtained after 35 cycles of amplification, then the specific value was not considered. The expression values of *PYK44* was multiplied by 10 due to its low expression. Moreover, the amplicon sequencing of this set of pyknons in several cell lines and commercial RNAs was also performed as quality control (data not shown).

### 2.6. Statistical Analysis

All the statistical analyzes were performed using R-environment (R Core Team, 2019) with Bioconductor packages (Huber et al., 2015) and SPSS software (SPSS for Windows version 24, SPSS Inc., Chicago, IL, USA). The relationship between the relative expression levels of selected pyknons in cancer vs. normal or paired groups was assessed using the Mann–Whitney *U*-test, and *P* ≤ 0.05 was significant. The area under the receiver operating characteristic curve (ROC), sensitivity, and specificity were obtained using the ROCR package (Sing et al., 2005). Association of clinicopathological and molecular features with pyknon expression was evaluated using Fisher's exact test. In these associations, a pyknon was dichotomized as high/low expression values according to median values of expression as a threshold. The overall survival (OS) interval was defined from diagnostic date to the time of the first event or the date on which data were censored, according to the method of Kaplan-Meier, and the curves were compared with the use of the log-rank test using survival package (Therneau and Grambsch, 2000; Therneau, 2015).

## 3. Results

The molecular status is summarized in Table 1. It was observed a frequency of 4.1% (3/73) of MSI-H cases. Moreover, 57.5% (42/73) of cases showed *APC* mutations, 50.7% (37/73) to *TP53* mutations, 50.7% (37/73) to *KRAS* mutations, 9.6% (7/73) to *PIK3CA* mutations, 6.8% (5/73) to *FBXW7* mutations, and none exhibited *BRAF* mutations (Table 1).

### 3.1. Pilot Cohort for Pyknon Candidate Selection

In order to validate the specificity of each PCR product and to technically select the best pyknons, i.e., those presenting ≥ 90% amplification efficiency and with potential for differential expression in colorectal cancer, a pilot study using 12 previously reported (*PYK10*, *PYK14*, *PYK17*, *PYK26*, *PYK27*, *PYK40*, *PYK41*, *PYK42*, *PYK43*, *PYK44*, *PYK83*, and *PYK90*) (Rigoutsos et al., 2017), was performed in 20 paired tumor/normal tissue cases (Supplementary Figure 1). Based on these results, some pyknons were excluded from further analyses due to the low number of samples amplified (*PYK14*, *PYK26*, *PYK27*, *PYK41*, and *PYK43*) (Supplementary Figure 1). *PYK90* showed no amplification in all samples analyzed (Supplementary Figure 1), whereas *PYK40* presented high levels of amplification but no difference between samples and controls (*p* = 0.81) (Supplementary Figure 1).

Therefore, five pyknons (*PYK10*, *PYK17*, *PYK42*, *PYK44*, and *PYK83*) could be detected in the most of samples, showed a *P*-value ≤ 0.1 in the pilot results (Supplementary Figure 1) and, consequently, were used for further evaluation in an expanded cohort of 73 CRC cases.

### 3.2. Validation Cohort: *PYK10, PYK17, PYK42, PYK44*, and *PYK83* Are Differentially Expressed in CRC Patients

**Figure 1:**
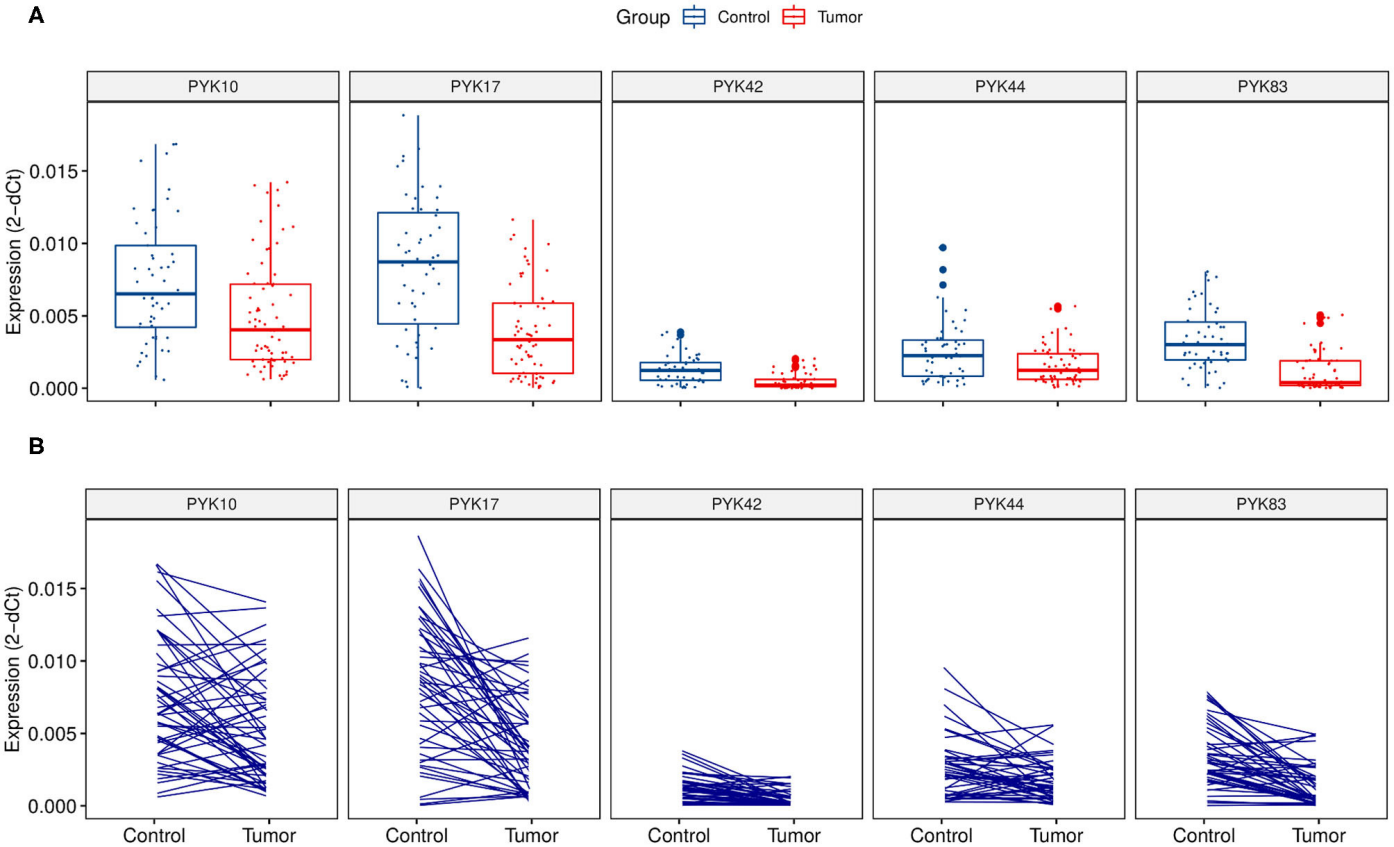
Relative pyknon expression in CRC samples by quantitative real-time PCR. **(A)** Expression and distribution of the five selected pyknons between 73 CRC patients and 52 normal samples. **(B)** Expression and distribution of pyknons of 52 paired samples. Y-axis values represent the ratio of each pyknon to the average of *GAPDH*, *U6*, and *ACTB*. The relative expression values of *PYK44* were multiplied by 10 due to its low expression.

In the validation cohort, RT-qPCR results confirmed that all five (*PYK10*, *PYK17*, *PYK42*, *PYK44*, and *PYK83*) pyknons showed a significant down-regulation between normal (*n* = 52) vs. tumor groups (*n* = 73) and also in the paired analysis (Figure 1 and Table 2). Furthermore, we performed the ROC curve analysis for sensitivity and specificity of the five pyknons analyzed, and observed significant results for *PYK17* (*p* = 1.26E-06, AUC = 0.77), *PYK42* (*p* = 2.65E-07, AUC = 0.78), and *PYK83* (*p* = 1.30E-09, AUC = 0.83) (Supplementary Figure 2). Even in paired sample analysis, all of them presented more than 0.75 of AUC and ≥70% of specificity (Table 2).

**Table 2:** Receiver operating characteristic (ROC) associated values for pyknons expression levels dichotomized (high/low) obtained from 73 colorectal cancer patients when compared with 52 normal tissue (global analysis) and 52 cancer/normal samples (paired analysis).

****Pyknon ID****	****Cutoff value****	****Sensitivity (%)****	****Specificity (%)****	****AUC****	*****P*****-value****
**Global analysis**					
*PYK10*	0.0055	64	62	0.67	0.002
*PYK17*	0.0065	67	80	0.77	3.04E-06
*PYK42*	0.0005	76	72	0.78	2.08E-06
*PYK44*	0.0002	63	66	0.64	0.005
*PYK83*	0.0020	74	82	0.83	3.72E-09
**Paired analysis**					
*PYK10*	0.0055	63	61	0.64	0.01
*PYK17*	0.0065	68	76	0.75	1.83E-06
*PYK42*	0.0006	74	68	0.76	1.61E-05
*PYK44*	0.0002	60	61	0.62	1.38E-05
*PYK83*	0.0020	74	77	0.79	4.97E-07

Next, we associate the differentially expressed pyknons with the patients' clinical and molecular features (Table 3). The dichotomized pyknon expression values showed that high *PYK17* expression was associated with higher histologic grade (*p* = 0.035), low *PYK44* expression was associated with higher clinical staging (*p* = 0.016) and high *PYK10* was associated with the presence of *TP53* mutations (*p* = 0.029) (Table 3). No significant association was observed for the *PYK42* and *PYK83* pyknons (Table 3).

**Table 3:** Association of pyknons expression levels (high/low) and colorectal patients' clinicopathological and molecular features (*n* = 73).

****Characteristics****	*****PYK10*****		*****P*****-value****	*****PYK17*****		*****P*****-value****	*****PYK42*****		*****P*****-value****	*****PYK44*****		*****P*****-value****	*****PYK83*****		*****P*****-value****
	****High****	****Low****		****High****	****Low****		****High****	****Low****		****High****	****Low****		****High****	****Low****	
**Age group**															
<68 years	19	23	0.325	14	27	0.794	15	21	0.061	16	22	0.801	10	26	1.00
≥ 68 years	9	19		8	19		5	22		10	16		8	18	
N/A	3			5			10			9			11		
**Gender**															
Female	9	23	0.087	7	25	0.119	7	23	0.189	9	18	0.440	7	20	0.780
Male	19	19		15	21		13	20		17	20		11	24	
N/A	3			5			10			9			11		
**Primary disease site**															
Cecum	0	1	0.947	1	0	0.621	0	1	0.216	1	0	0.693	0	1	0.187
Descendent colon	0	1		0	1		0	1		0	0		0	1	
Sigmoid colon	10	15		9	15		9	14		10	13		3	18	
Rectosigmoid junction	4	8		3	9		6	6		4	8		6	6	
Rectum	14	17		9	21		5	21		11	17		9	18	
N/A	3			5			10			9			11		
**Clinical staging (at diagnosis)**															
0/I	2	13	0.085	3	12	0.408	3	12	0.644	1	13	0.016^*^	3	12	0.553
II (a-c)	17	20		13	22		11	21		18	16		10	23	
III (b-c)	7	8		6	9		5	9		6	7		5	7	
IV (b)	2	1		0	3		1	1		1	2		0	2	
N/A	3			5			10			9			11		
**Primary tumor (T)**															
Tis/T1	3	8	0.473	2	8	0.148	1	9	0.227	3	7	0.118	1	7	0.174
T2	4	10		4	10		4	10		2	11		3	11	
T3	17	21		11	26		11	21		18	17		10	24	
T4 (a, b)	4	3		5	2		4	3		3	3		4	2	
N/A	3			5			10			9			11		
**Regional lymph nodes (N)**															
N0	18	31	0.278	16	31	1.00	31	13	0.544	27	18	0.783	13	32	0.754
N1/N2	10	9		6	13		10	7		10	8		5	10	
N/A	5			7			12			10			13		
**Distant metastasis (M)**															
M0	26	41	0.560	22	43	0.546	19	42	0.538	25	36	1.00	18	42	1.00
M1a	2	1		0	3		1	1		1	2		0	2	
N/A	3			5			10			9			11		
**Histologic grade**															
I	1	0	0.131	1	0	0.035^*^	1	0	0.249	1	0	0.133	0	1	0.698
II	24	41		18	45		17	41		22	37		16	41	
III	3	1		3	1		2	2		3	1		2	2	
N/A	3			5			10			9			11		
**Radiotherapy**															
Yes	3	4	1.00	3	4	0.393	0	6	0.273	2	3	0.489	2	4	0.611
No	25	37		19	41		20	36		24	35		16	39	
N/A	4			6			11			9			12		
**Adjuvant chemotherapy**															
Yes	14	28	0.141	10	16	0.594	8	16	1.00	14	11	0.068	8	15	0.567
No	14	13		12	29		12	26		12	27		10	28	
N/A	4			6			11			9			12		
**MSI status**															
MSS+MSI-L	26	39	0.281	18	45	0.216	18	41	1.00	23	37	0.557	17	40	0.381
MSH	0	3		2	1		1	2		2	1		0	3	
N/A	5			7			11			10			13		
***APC*** **mutation**															
Yes	19	21	0.736	14	24	0.460	14	22	1.00	17	21	0.488	11	26	1.00
No	4	6		2	8		3	5		3	7		2	7	
N/A	23			25			29			25			27		
***TP53*** **mutation**															
Yes	20	15	0.029^*^	13	20	0.322	14	18	0.315	16	18	0.338	12	20	0.072
No	3	12		3	12		3	9		4	10		1	13	
N/A	23			25			29			25			27		
***KRAS*** **mutation**															
Yes	14	22	1.00	9	26	0.431	8	22	0.588	14	24	0.195	6	26	0.093
No	12	20		11	20		11	21		11	14		11	17	
N/A	5			7			11			10			13		
***KRAS*** **mutation**															
Yes	1	6	0.107	1	5	0.648	1	4	0.634	2	4	1.00	1	4	1.00
No	22	21		15	27		16	23		18	24		12	29	
N/A	23			25			29			25			27		
***FBXW7*** **mutation**															
Yes	4	1	1.67	2	3	1.00	3	2	0.359	3	2	0.636	2	2	0.565
No	19	26		14	29		14	25		17	26		11	31	
N/A	23			25			29			25			27		

* *N/A, Not available; *BRAF* mutation: no statistic was calculated since all samples are negative for the mutation. The bold values are the significant values*.

### 3.3. Lower Tumor PYK44 Expression Is Associated With a Worse Prognosis

**Figure 2:**
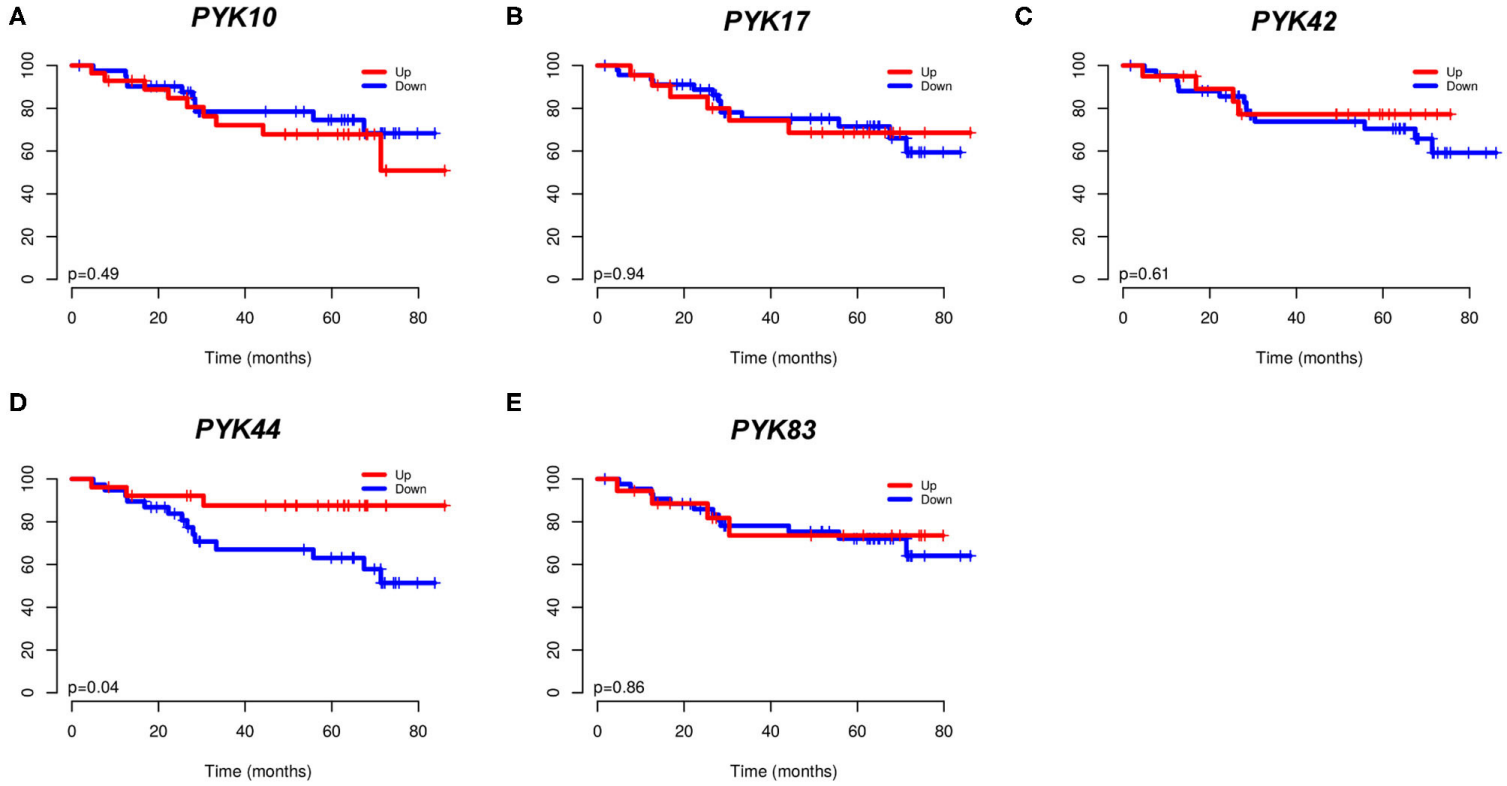
Kaplan-Meier curves from 73 CRC patients. In **(A)** *PYK10*; **(B)** *PYK17*; **(C)** *PYK42*; **(D)** *PYK44*; and **(E)** *PYK83*. The expression was dichotomized (high/low) according to a cutoff value corresponding to the median value of all patients.

Furthermore, we evaluated the association of pyknon expression status (high/low) with overall patient survival (OS) (Figure 2). We found that low levels of *PYK44* were significantly associated with inferior OS (*p* = 0.04, Figure 2D). Although its relative expression values are lower than the other pyknons, *PYK44* presented a fold change of 1.6 between normal and tumor samples.

## 4. Discussion

The present study intended to continue a recent study that reported the role of pyknons, a novel type of ncRNAs, in colorectal tumorigenesis (Rigoutsos et al., 2017). We evaluated the relative expression levels of 12 pyknons motifs (*PYK10*, *PYK14*, *PYK17*, *PYK26*, *PYK27*, *PYK40*, *PYK41*, *PYK42*, *PYK43*, *PYK44*, *PYK83*, and *PYK90*) in a series of 73 CRC Brazilian patients compared to 52 normal tissue counterpart and associated these results with clinicopathological, molecular and genetic ancestry features. We observed that *PYK10*, *PYK17*, *PYK42*, *PYK44*, and *PYK83* exhibited significantly lower expression in tumors compared with adjacent normal tissue. Moreover, we found that high *PYK10* expression was associated with *TP53* mutations, and *PYK44* downregulation was associated with poor prognosis in CRC.

The present results do not validate the results obtained by Rigoutsos et al. (2017), which report an overall overexpression of pyknons in tumor tissue. Notably, the authors reported that *PYK14*, *PYK40*, *PYK41*, *PYK44*, and *PYK90* were significantly upregulated (Rigoutsos et al., 2017). The *PYK90* was also found to be upregulated in gastric cancer (Youn et al., 2019). In our series, the *PYK44* was significantly downregulated in tumor tissue, the *PYK40* did not change between tumor and normal tissue, *PYK41* exhibited a shallow expression level, and *PYK90* had an absence of expression. However, Rigoutsos et al. (2017) did not report expression level alterations between the groups evaluating either association of *PYK44* with OS. Despite the use of the same primer set and methodology, some reasons can explain these results. In the present study, we performed a paired analysis of tumor and adjacent normal tissue, at variance with Rigoutsos et al. (2017), in which tumor and normal tissue were derived from distinct patients. Moreover, the distinct geographic location of patients and distinct exposure could explain such differences since it has been suggested that race disparities could contribute to ncRNA expression (Huang et al., 2011). A previous study by our group characterized the genetic ancestry in the same study patients, showing the prevalence of European ancestry in our samples in 83.1% of the samples (Dos Santos et al., 2019). Also, Dragomir et al. (2019) recently showed downregulation of several pyknons after evaluating non-infection complications of splenectomy, including *PYK10*, *PYK14*, and *PYK17*. These findings show that pyknon expression could change in another context.

In the present study, we found that the downregulation of the *PYK17* was associated with histologic grade II, suggesting a putative biological role for this pyknon. Interestingly, we found an association between high *PYK10* with *TP53* mutation. This finding is in line with previous studies since several non-coding RNAs are reported as associated with the regulation of cancer pathways, including the p53 pathway, a master regulator of the cell cycle, and survival (Huarte et al., 2010). A recent study reported that genes regulated by *FLANC*, a novel lncRNA co-localized with a pyknon motif, could regulate genes that are key components of the p53 pathway (Pichler et al., 2020).

Rigoutsos et al. (2017) also reported down-regulation of *PYK42* and associated the lower expression of this pyknon with the MSI-H phenotype, suggesting that the *PYK42* expression could be associated with most aggressive colorectal tumors. Likewise, we also observed down-regulation of *PYK42* in the tumor when compared with normal adjacent tissue; however, only three cases exhibited MSI-H, hampering any meaningful conclusion.

Our results also showed down-regulation of all pyknons analyzed. These molecules are new players between the non-coding regulation molecules, and further studies are necessary to understand their modulation patterns and functional role (Dragomir et al., 2020). Considering that pyknons *loci* show sequence similarity with several known miRNAs that are frequently located in fragile sites of the genome associated with cancer (Calin et al., 2004; Durkin and Glover, 2007; Bartel, 2009; Hansji et al., 2014; Ling et al., 2015), recent studies have suggested that genomic events in these regions could lead to the down-regulation of miRNAs in a broad range of tumors (Lu et al., 2005; Garzon et al., 2006; Juan et al., 2010). Additionally, down-regulation of long non-coding RNAs is reported in CRC, and therefore other non-coding RNAs, such as pyknons, could show this behavior as well (Schetter et al., 2012; Mohammadi et al., 2016).

The deeper understanding of the possible regulatory role of pyknons as tumor suppressors or oncogenic non-coding RNAs is still in its infancy. Pyknons dysregulation (high or lower expression in tumors) could also be associated with transcriptional and epigenetic regulation, imprinting, splicing, subcellular transport, a scaffold for protein–protein interactions, kinase function regulation of metabolic checkpoints, following the same pattern observed in other long non-coding RNAs (Inamura, 2017). Finally, new evidence has pointed out the role of pyknons to locate functional other classes of non-coding RNAs, as a method to distinguish between functional and “junk” transcripts (Danis and Széll, 2018).

Concluding, we reported the expression profile of pyknons motifs and showed its significant downregulation in tumor tissue when compared with the normal counterpart, and the association of *PYK44* lower expression with worse patient outcomes. Further studies are needed to extend and validate these findings and determine their clinical-pathological impact.

## Data Availability Statement

The raw data supporting the conclusions of this article will be made available by the authors, without undue reservation.

## Ethics Statement

The studies involving human participants were reviewed and approved by Barretos Cancer Hospital Ethical Committee. The patients/participants provided their written informed consent to participate in this study.

## Author Contributions

AE and WM were responsible for collecting data, sample preparation, and qRT-PCR experiments. WD and GB were responsible for MSI, ancestry, and sequencing experiments. DG and CS-N were the medical doctors involved in the selection of samples. AE analyzed the data and wrote the original paper in English. GC was responsible for the initial idea, protocols, and discussion of the results. RR supervised the study, made revisions, edited the use of language, and ultimately wrote the final version of the manuscript. All authors contributed to the article and approved the submitted version.

## Conflict of Interest

The authors declare that the research was conducted in the absence of any commercial or financial relationships that could be construed as a potential conflict of interest.

## Supplementary Material

Supplementary Table 1 Primer sequences used for quantitative real time PCR in this study.

Supplementary Figure 1 Pyknon's relative expression in 20 paired CRC samples of the pilot study was done by quantitative real-time PCR. Y-axis values represent the ratio of each pyknon to the average of *GAPDH*, *U6*, and *ACTB*. The relative expression values of *PYK26*, *PYK27*, *PYK41*, *PYK43*, *PYK44*, and *PYK90* were multiplied by ten due to its low expression.

Supplementary Figure 2 Receiver operating characteristic (ROC) curve for dichotomized expression values of pyknons obtained from 73 CRC patients and 52 normal samples. The area under the ROC curve (AUC) is shown to **(A)** *PYK10*; **(B)** *PYK17*; **(C)** *PYK42*; **(D)** *PYK44* and **(E)** *PYK83*.
