# Supplementary material for: Pyknon-Containing Transcripts Are Downregulated in Colorectal Cancer Tumors, and Loss of PYK44 Is Associated With Worse Patient Outcome
Source: Front Genet. 2020 Nov 12;11:581454. doi: 10.3389/fgene.2020.581454 (PMC7693444; doi:10.3389/fgene.2020.581454)
Supplement: Supplementary Table 1 — Primer sequences used for quantitative real time PCR in this study. [file Table_1.DOCX]

**Supplementary Table I. Primer sequences used for quantitative real time PCR in this study.**

| **Pyknon ID** | **Primer sequence 5'-3'** |
| --- | --- |
| *PYK10* (Forward) | AAGTGGGCTCTGAGTGTGGT |
| *PYK10* (Reverse) | CCTGACTTCGTGATCCACCT |
| *PYK14* (Forward) | CCCTCCTCTTCTTTAGTGAATCC |
| *PYK14* (Reverse) | CAGCTCACTGCAGTCTTAACCTC |
| *PYK17* (Forward) | CCTCCCTGTTAAGCCCAGTT |
| *PYK17* (Reverse) | ACTCCTTTGTGCAAGCCAGA |
| *PYK26* (Forward) | TGCGTGACGATAACAATATGC |
| *PYK26* (Reverse) | CACCTGGGCCTCTCAAAGT |
| *PYK27* (Forward) | GTGGCCACACTGATTCTAGC |
| *PYK27* (Reverse) | CCTCCCTCTTCAACCTCTCA |
| *PYK40* (Forward) | GCAGCCTCAACCTCCTGA |
| *PYK40* (Reverse) | GGAGTTGAGGACCAGCCTAGA |
| *PYK41* (Forward) | CTGGTCTTAAACTCCTGACCTTC |
| *PYK41* (Reverse) | AGAATCCTGACATGAGATCATCC |
| *PYK42* (Forward) | TTCTGAAAGTCACAAAGCTGGA |
| *PYK42* (Reverse) | TTCACCATGTTTGCCAGGAT |
| *PYK43* (Forward) | AAGCAACATTGGGTACTTCTTCA |
| *PYK43* (Reverse) | TGCTGACCTCAAGCAATTCA |
| *PYK44* (Forward) | TCTCCTGACCTCATGATCCA |
| *PYK44* (Reverse) | CACAGTGCTCAGCCAAAATG |
| *PYK83* (Forward) | GTGAATCCAGGCACCTTCTG |
| *PYK83* (Reverse) | CGTTCCTCAATAAAGCTGTGC |
| *PYK90* (Forward) | CTCGCTTCCAACAGAGAAGG |
| *PYK90* (Reverse) | CGGGCTTGATCGTACAATTT |
