# Supplementary figures and images for: Pyknon-Containing Transcripts Are Downregulated in Colorectal Cancer Tumors, and Loss of PYK44 Is Associated With Worse Patient Outcome
Source: Front Genet. 2020 Nov 12;11:581454. doi: 10.3389/fgene.2020.581454 (PMC7693444; doi:10.3389/fgene.2020.581454)

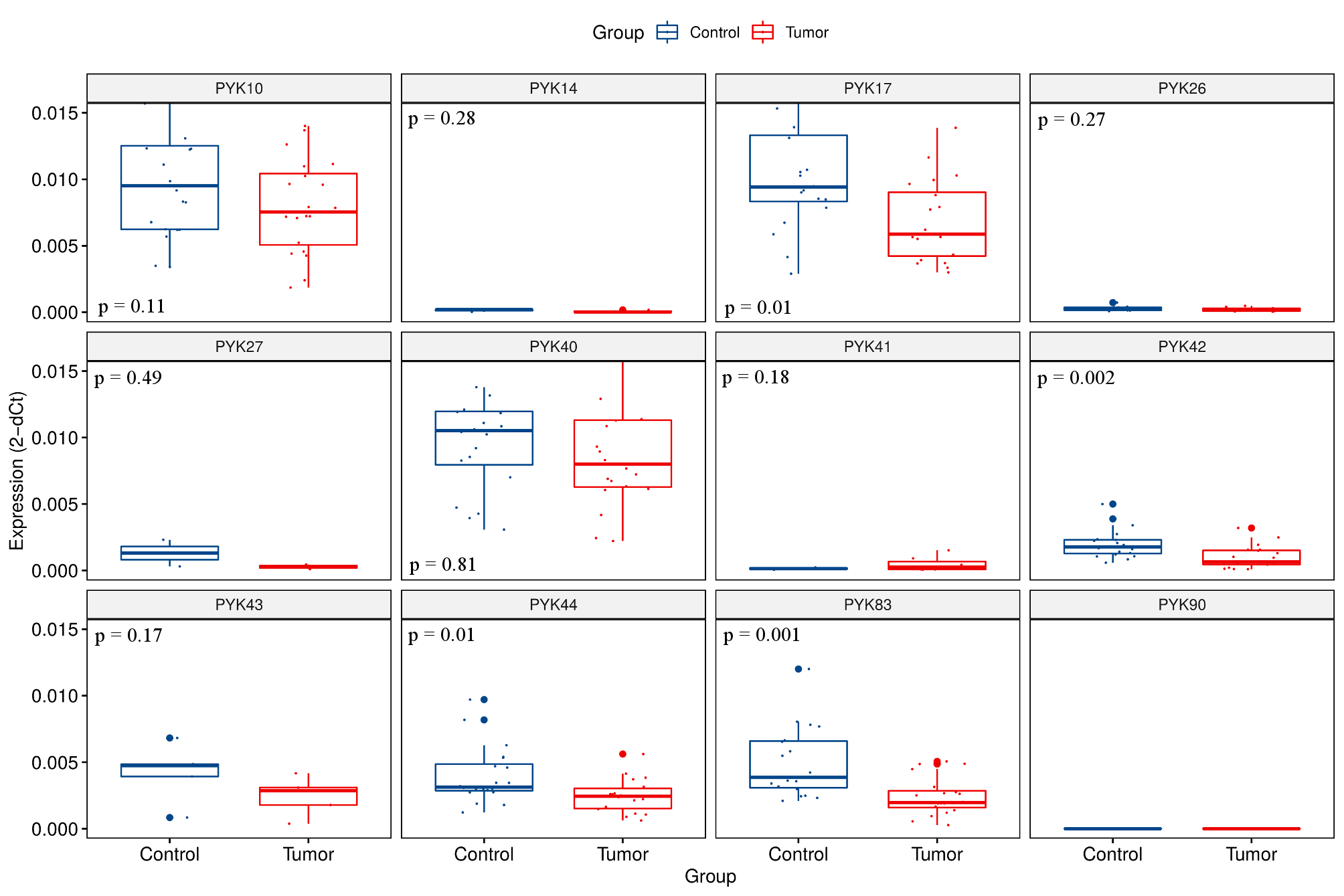

Supplement: Supplementary Figure 1 — Pyknon's relative expression in 20 paired CRC samples of the pilot study was done by quantitative real-time PCR. Y-axis values represent the ratio of each pyknon to the average of GAPDH, U6, and ACTB. The relative expression values of PYK26, PYK27, PYK41, PYK43, PYK44, and PYK90 were multiplied by ten due to its low expression. [file Image_1.TIFF]

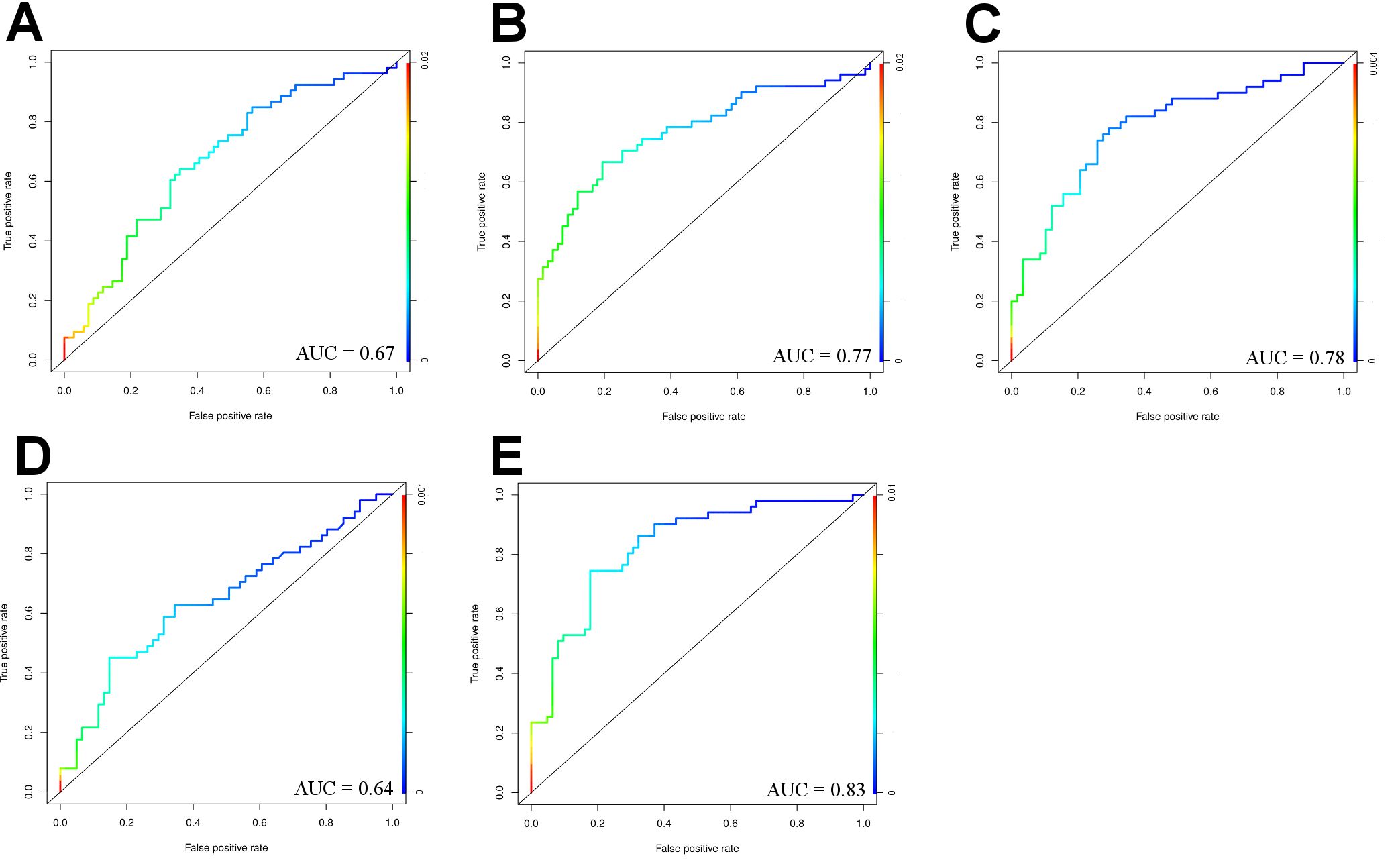

Supplement: Supplementary Figure 2 — Receiver operating characteristic (ROC) curve for dichotomized expression values of pyknons obtained from 73 CRC patients and 52 normal samples. The area under the ROC curve (AUC) is shown to (A) PYK10; (B) PYK17; (C) PYK42; (D) PYK44 and (E) PYK83. [file Image_2.TIFF]
